# Alkali-Ion-Crown Ether in Art and Conservation: The Applied Bioinorganic Chemistry Approach

**DOI:** 10.1155/S1565363304000020

**Published:** 2004

**Authors:** Uwe Hilfrich, Harold Taylor, Ulrich Weser

**Affiliations:** Anorganische Biochemie Physiologisch-Chemisches Institut der Eberhard-Karls Universität Tübingen Hoppe - Seyler- Str. 4 Tübingen D-72076 Germany

## Abstract

Dried varnish is rich in many ester moieties, which may be broken down into small, soluble compounds by esterase activity or alkaline hydrolysis. Two methods for varnish removal have been developed, including the treatment of either lipase or RbOH / PEG-400 crown ether which allow aged oil varnishes or paint coverings to be removed or thinned. These techniques are designed to proceed in a controlled manner without damaging lower paint or base layers. Unfortunately, lipase did not react with the aged ester groups of dried linseed oil varnish. Surprisingly, the varnish came off in the presence of Tris buffer alone which, in addition, formed reactive metal complexes. A better choice was the use of high M_r_ alkali ion polyethylene glycol–400 (PEG-400) crown ether type chelates. PEG-400 complexes alkali ions including rubidium and other alkaliions impeding the diffusion of their basic counter ions into lower varnish or paint layers. Possible migration of alkali metal ions into the paint layer during alkaline varnish removal was determined by labelling the cleansing solutions with ^86^Rb. Fortunately, varnish is degraded on the surface only. Lower paint or varnish layers are not attacked even if chemically similar to the varnish or over painting to be removed as virtually no ^86^Rb was detected on the paint surface.


### Alkali-Ion-Crown Ether in Art and Conservation: The Applied Bioinorganic Chemistry Approach

Uwe Hilfrich, Harold Taylor, Ulrich Weser*

*Anorganische Biochemie, Physiologisch-Chemisches Institut der Eberhard-Karls Universität Tübingen, Hoppe - Seyler - Str. 4 D - 72076 Tübingen, Germany*

### ABSTRACT

Dried varnish is rich in many ester moieties, which may be broken down into small, soluble compounds by esterase activity or alkaline hydrolysis. Two methods for varnish removal have been developed, including the treatment of either lipase or RbOH / PEG-400 crown ether which allow aged oil varnishes or paint coverings to be removed or thinned. These techniques are designed to proceed in a controlled manner without damaging lower paint or base layers. Unfortunately, lipase did not react with the aged ester groups of dried linseed oil varnish. Surprisingly, the varnish came off in the presence of Tris buffer alone which, in addition, formed reactive metal complexes. A better choice was the use of high $M_r$ alkali ion polyethylene glycol – 400 (PEG-400) crown ether type chelates. PEG-400 complexes alkali ions including rubidium and other alkali-ions impeding the diffusion of their basic counter ions into lower varnish or paint layers. Possible migration of alkali metal ions into the paint layer during alkaline varnish removal was determined by labelling the cleansing solutions with $^{86}Rb$. Fortunately, varnish is degraded on the surface only. Lower paint or varnish layers are not attacked even if chemically similar to the varnish or over painting to be removed as virtually no $^{86}Rb$ was detected on the paint surface.

**Keywords:** varnish removal, lipase, Cu (II) -Tris, linseed oil, RbOH / PEG-400 crown ether.

### 1. INTRODUCTION

Varnish is the final, sealing layer of a painting, imparting depth, luminance, gloss or matte while protecting it from mechanical as well as atmospheric influences.

---

* *Corresponding author:* Tel.: +497071 – 295564; Fax.:+497071 - 295564

e-mail: ulrich.weser@uni-tuebingen.de (U. Weser)

Internet: http://www.pci.chemie.uni-tuebingen.de/weser/weser.html

The varnish layer itself is the most exposed layer of a painting and should in cases of age-related discoloration be easily removable. Due to its composition, large surface area and small depth, varnish layers react to mechanical and environmental influences with more or less extreme changes including, yellowing, cross linking and crazing. These changes to a painting's shape and tonal balance ultimately necessitate the removal of the varnish layer.

Varnishes for paintings were based primarily on drying oils until the late Middle Ages. Recipes describing such varnishes can be found in the tractates of medieval painters /1/. The simple process of boiling certain oils or boiling them with natural resins prepared these varnishes. The resulting highly viscous, dark liquids were applied to the paintings by a brush or manually. Occasionally, pure, unadulterated oil was applied as a varnish. Upon exposure to air, a varnish will dry slowly over a period of months or years. This drying process can be accelerated by sunlight or dryers (siccatives). Whereas lead white (basic lead carbonate) or lead oxides were traditionally used, modern dryers contain lead, manganese or cobalt oleates or resinates.

The discovery of distillation in the late Middle Ages led to the application of turpentine oil and as resin solvents resulting in improved varnishes. These resin essence varnishes dry faster and are considerably lighter than oil varnishes – very likely the reason they have largely replaced oil varnishes in modern painting. There is a chemical relationship between aged linseed and resinous varnishes in that they represent in part or full a polyester type structure.

While resin essence varnishes are usually easily removable with organic solvents, aged oil varnishes are resistant to these solvents. Varnishes always cover paint layers, which are, of their nature, susceptible to solvents. Selective removal of varnish becomes almost impossible if the paint binder is of similar or greater solubility than the covering varnish. A similar situation occurs when trying to remove over-paintings of oil paints, if the over-painting was applied directly onto the original layer. The chemistry of aged oil paint and varnishes, as well as the gentle removal thereof, are accordingly of special interest in the conservation of our cultural heritage of paint; current technology cannot solve this problem chemically.

A promising new technique for the cleaning of painting surfaces was introduced employing lipase. The lipase based removal of aged oil varnishes was claimed to be both gentle and protective /2,3/. Alternatively, a new concept in alkaline varnish removal was developed taking into account the reactivity of strong bases and larger counter cations. The large relative molecular mass solvent PEG-400 was expected to form complexes with alkaline ions as macromolecular crown ethers impeding the diffusion of their basic counter ions into lower varnish or paint layers. These two biochemical approaches employing enzymic and bioinorganic methods in the cleaning of paint surfaces will be comprehensively dealt with.

### 2. EXPERIMENTAL

### 2.1. Materials

### *2.1.1. Chemicals*

Radioactive rubidium-86 (half-life: 18.7 days; type of decay: $\beta^-$; $\beta^-$ energy (max.): 0.69 MeV (8.8%) / 1.77 MeV (91.2%); $\gamma$ radiation 1.077 MeV (8.8%)) was delivered as an aqueous rubidium chloride solution from Amersham Pharmacia Biotech, code RGS.2. The radioactivity was 37 - 185 MBq/ml or 1 - 5 mCi/ml. $^{14}C$ polyacrylic acid was generously donated by Dr. Eugen Barbu, School of Pharmacy and Biomedical Sciences, University of Portsmouth, Hampshire, UK.

Window glass plates, length: 18 cm; width: 10 cm, thickness: 3 mm, were obtained from Glaserei Härle, Tübingen, Germany.

All other reagents including rubidium hydroxide were of the highest purity available and obtained from Sigma-Aldrich, Steinheim Germany.

### *2.1.2. Buffer gel and enzymic cleaner*

300 mg hydroxymethylpropylcellulose were allowed to swell in 20 ml water for one hour. A stiff gel was obtained after stirring. The gel was mixed with 10 ml 0.1 M Tris buffer (adjusted with HCl to pH 8.4 or pH 7.7) and 0.02 ml Triton X- 100.

The enzyme gel was prepared by dissolving 10 mg / 1 (= 8600 u/ml) lipase VII (E.C. 3.1.1.3 from Candida cylindracea, Sigma L-1754) in the buffer gel.

### *2.1.3. Gel chromatography*

Gel chromatography of RbOH-PEG-400 complex was carried out using Bio-Gel P-2 (100-200 mesh) from Bio-Rad Laboratories, Richmond, California. 10 ml of a mixture of 1 mol/l RbOH and a 28 fold excess of PEG-400 were passed through a column 1.3 x 43 cm. Quantification of Rb-ions was performed using atomic emission spectroscopy at 780.023 nm on a Zeiss M4 Q III unit equipped with a PM QII detector.

### *2.1.4. Reaction of Tris buffer solutions with copper pigments*

Samples of dried linseed oil paint film containing a copper pigment (malachite or verdigris) were scraped off with a scalpel and placed into a glass dish containing aqueous 0.05 M pH 8.4 tris buffer solution. Within 20 minutes the solution turned light blue, attributable to the formation of tris copper complexes. The colour became dark blue overnight. Removal of the buffer solution showed the paint samples had turned brown and deteriorated.

### *2.1.5. Paintings*

An authentic fragment of a late 17th century oil painting on canvas was cleansed employing the crown-ether method at the Courtauld Institute of the History of Art, London. The partial cleaning of the painting 'Die Fischhändlerin' 1874 ('pseudo Frans Hals') was possible at the Institut für Restaurierungs und Konservierungs-wissenschaften, Köln.

### 2.2. Preparations

### *2.2.1. Varnish*

*Type 1:* Fresh cold pressed linseed oil (Kremer, Aichstetten) for direct use as a varnish. *Type 2:* Linseed oil varnish accelerated drying in the presence of lead oxide.

The 0.5 g PbO are suspended in 100 ml fresh linseed oil. The suspension is heated to 220° for 2-3 hours under nitrogen while stirring is maintained. The dark brown varnish is decanted after cooling and stored under nitrogen.

Liquid varnishes are applied in a thin layer to cleansed glass plates (0.2 - 0.5 ml per 100 $cm^2$). The plates are then dried at 25°C. The drying rate may be increased by raising the temperature of the drying cabinet to a maximum of 40°C. To achieve complete and thorough hardening, as well as a minimum of similarity to aged varnishes, the varnish plates are subsequently stored for up to seven years. The time required for non-stick drying depends upon the type of varnish. Average times at 25°C required for non-stick drying of the various varnishes are:

- pure linseed oil: 2 – 3 weeks accelerated drying;
- linseed oil varnish in the presence of PbO: 3 – 4 days.

### *2.2.2. Model oil paint surfaces*

The corresponding pigments (Kremer, Aichstetten) are mixed with linseed oil until smooth. The resulting paint is applied to the meticulously cleansed glass plates and dried at 25°C. When thoroughly hardened (3 - 7 days), the paint layer is varnished as described in section 2.1. The plates are stored for several years.

### *2.2.3. Alkali-ion PEG-400 crown ether*

One gram of rubidium hydroxide is dissolved in 10 ml PEG-400 at 60 - 70°C. Alkaline solutions of PEG gradually turn brown due to oxidation. They should consequently be stored under exclusion of oxygen. RbOH in PEG-400 is applicable in cases of thick layers of grime. However, the surface should be neither porous nor contain craquelures. The varnish dissolves with an orange-brown color and can be removed with cotton swabs. The solution should be agitated slightly to improve the cleansing action by using a polypropylene spatula. The thinning of varnish must be monitored continuously. For safety purposes, a thin varnish layer should be left covering the paint. If required, the alkaline cleansing solution may be neutralized rapidly by the addition of polyacrylic acid in PEG-400.

Should the picture surface be soiled with the salts of small cations, a pre-treatment with an acidic cation exchanger or polyacrylic acid in PEG-400 is required. The surface should then be cleansed with pure PEG-400. After varnish removal or thinning, the cleanser should be removed with dry cotton swabs. The surface should then be cleansed with PEG-400. A gel containing polyacrylic acid in PEG-400 should be applied and incubated for 1 - 5 min. to neutralize possible alkaline remnants. Once the polyacrylic acid gel is removed, the surface of the painting should be treated step by step with the following solutions: PEG-400 / dodecanol 1:1, poly-(1,2-propanediol) 1000 and then PEG-4000. The solvent polarity will be reduced to the point at which the non-volatile polyethers can be removed completely in a final step by several treatments with iso-dodecane (Shellsol-T). The surface of the painting is then allowed to dry by iso-dodecane evaporation.

### 2.3. Analytical procedures

### *2.3.1. Back titration of aged varnish*

Three month aged layers of dried linseed oil varnish are scraped off from glass plates with a scalpel. The carboxylic acid content of the varnish is determined by maceration of 50 - 150 mg varnish in 10 ml sodium hydroxide (50mM) for 1 hour in an ultrasonic bath at 25°C. Upon this treatment, the sample solution is yellowish-orange and slightly turbid.

This solution is back titrated using 25mM sulfuric acid. The amount of sodium hydroxide consumed by saponification is the difference between the amounts of sulfuric acid theoretically and actually required for titration. The titration results of fresh linseed oil as a control and dried varnish are compared to ascertain that the former does not contain free carboxylic group and that the glycerol esters of the latter are not already hydrolysed by sodium hydroxide at 25°C. It could be shown that neither fresh linseed oil (type 1) nor linseed oil varnish with a lead siccative (type 2) contain significant amounts of free carboxylic acid or ester groups which can be hydrolysed at 25°C.

### *2.3.2. Infrared spectrometry*

FT-IR spectrometry of different linseed oil samples was performed on a Beckman Acculab 4 Spectrometer. Fresh and aged linseed oil were incorporated into a KBr-pill prior to the measurements.

### *2.3.3. Absorption studies on the paint surface using $^{86}$Rb-PEG400 crown ether and $^{14}$C-polyacrylate*

In order to locate $Rb^+$-ions in the course of alkaline varnish removal, the cleansing solution was enriched with $^{86}$Rb. Possible remaining rubidium ions on the paint surface could be determined in this way. The $^{86}$Rb-solution was diluted 1:1 with water and applied in the following experiments (stock solution).

The $^{86}$Rb stock solution (50 μl) was diluted with 500 μl of a mixture containing 1% triton X-100, 19% methanol and 80% water. An aliquot (200 μl) of this labelled solution was applied to a varnish plate and distributed evenly on a rectangular area of 50 cm$^2$ using a fine-haired, plastic brush. When dry, possible β-radiation at the varnish surface was determined using an automatic TLC-Linear Analyzer LB 2821, Berthold Technologies, Bad Wildbad, Germany. The apparatus allows measurements along a segment of the surface creating point-to-point radioactive emission profiles of the test plate.

The emission density of the calibration surface as determined by the detector was 900 ± 100 cpm (counts per minute; measured: 4500 ± 450 counts in 5 min.). The standard deviation of 10% results from a non-uniform distribution of the calibration solution. As with the $^{86}$Rb-PEG400 crown ether experiments $^{14}$C-polyacrylic acid was added to the polyacrylate neutralisation step and carbon-14 was monitored using the same experimental setup given in *2.2.3*.

Alkaline RbOH / PEG cleanser; an equimolar solution of RbOH in ethanol and an aqueous solution of RbOH in water, each of which were labelled with the same amount of $^{86}$Rb (50μl $^{86}$Rb stock solution and 500 μl of the solution to be tested), resulted in the same final concentration of $^{86}$Rb as in the calibration solution. The volume of these labelled solutions (200 μl), as well as the types and surface areas (50 cm$^2$) to which they were applied, were identical to those used for calibration. The rubidium concentration in all three solutions was identical. The application of 200 μl to a 50 cm$^2$ surface results in a rubidium density of

2.36 µmol/cm$^2$ or 900 cpm as determined in the calibration step. After 3 minutes the Rb($^{86}$Rb)-solution was wiped off using an analytical grade cellulose filter paper. Post-treatment of the paint surface following the Rb($^{86}$Rb)-PEG-crown ether cleansing was carried out stepwise with polyacrylate and two washes of PEG-400. Rb($^{86}$Rb)-ethanol treatment was followed by two ethanol washes. After the aqueous Rb($^{86}$Rb)OH treatment the paint surface was rinsed two times with water. In the case of $^{14}$C-polyacrylic acid, neutralisation experiments, aiming at possible surface absorption of $^{14}$C-polyacrylate following the cleansing process, were carried out as described above.

### 3. RESULTS AND DISCUSSION

### 3.1. Chemistry and structure of aged linseed oil varnish

Currently, one of the major problems in the chemical removal of dried oil varnishes and paints is their unknown chemical composition. Any reliable conservation concept would have to be based on a thorough and complete knowledge of the chemical nature of varnish and binder. Although sound in its scientific elucidation on the mechanism of oxidative hardening of 'drying oils' by Kaufmann /4/, no further genuine advance in this uncontrolled mechanism is available /5/.

Linseed oil consists primarily of various triglycerides containing linolenic (cis, cis, cis-9, 12, 15-octadecatrienic acid) and linoleic acid (cis, cis-9, 12-octadecadienic acid). Current reaction mechanism postulates that the drying of linseed oil begins with an autoxidation of the glyceride fatty acids within the oil. In addition polyunsaturated fatty acid residues are progressively oxidized leading to uncontrolled cross linking and carboxylic acid residues (Fig. 1).

The end product is called linoxin and its structure remains unknown. In the presence of oxygen, the state of the oil molecule progresses from a liquid via semi-solid intermediates to a firm, elastic layer. This layer can store pigments, thus 'binding' them to the support. In an attempt to obtain some structural information the drying process of linseed oil was monitored by infrared spectrometry. The spectrum of dried linseed oil differs only slightly from that of fresh oil. The numbers of double bonds decrease in the course of drying. The vibrational bands at 3011 cm$^{-1}$ (alkenyl H-valence vibrations), 1652 cm$^{-1}$ (C=C-valence vibrations) and 722 cm$^{-1}$ (cis olefines, deformational vibrations) diminish or disappear completely (Table 1).

The spectrum of dried linseed oil shows an intense OH-valence band at 3450 cm$^{-1}$ resulting either from absorbed water or from hydrogen-bonded OH-groups. The vibrational bands of the ester groups in linoxin are essentially unchanged compared to fresh linseed oil (C=O-valence vibrations at 1745 cm$^{-1}$ and C-O-valence vibrations at 1163 cm$^{-1}$). Consequently, the drying process does not affect the glyceride ester function.

An emulsion of fresh linseed oil is extraordinarily resistant to hydrolysis in the presence of cold sodium hydroxide. Significant hydrolysis of linseed or similar vegetable oils requires prolonged boiling in strong alkalines. By way of contrast, the carboxylic acid groups of slowly dried linseed oil or the fast dried version in the presence of PbO usually called linoxin react effectively with sodium hydroxide.

Comparative back titration experiments using both fresh and aged linseed oil were performed. Each component was treated with cold, aqueous sodium hydroxide. Back titration was performed using sulphuric

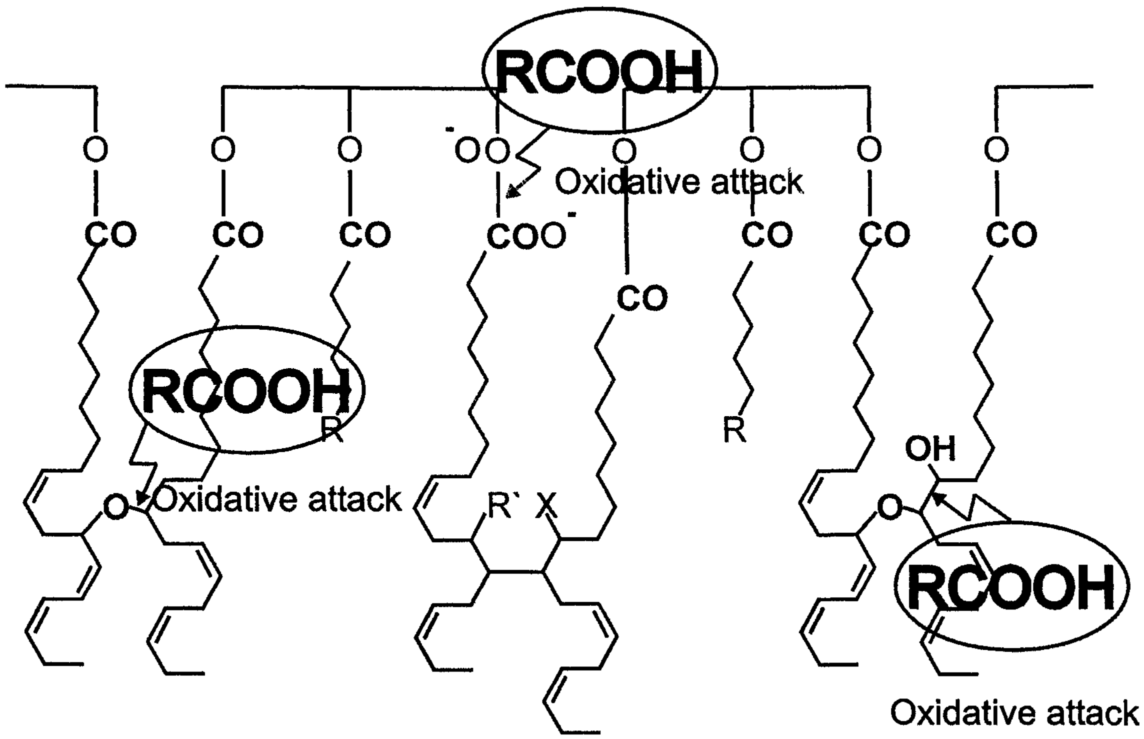

**Fig. 1**: Proposed sketch of oxidized layers of dried linseed oil.

**Table 1**

Characteristic IR-vibrational bands of different linseed oil preparations.

| band | type | linseed oil, fresh $\nu$ (cm$^{-1}$) | linseed oil, fresh rel.intensity (%) | linseed oil, dried $\nu$ (cm$^{-1}$) | linseed oil, dried rel.intensity (%) |
|---|---|---|---|---|---|
| 1 | O–H (H-bond) | — | — | 3452 | (44) |
| 2 | 2 C=O (overtone) | 3470 | (10) | -obscured- | |
| 3 | alkenyl-H | 3011 | (84) | — | — |
| 4 | 1 alkyl-H ($CH_2$) | 2926 | (100) | 2928 | (99) |
| 5 | 2 alkyl-H ($CH_2$) | 2854 | (96) | 2855 | (76) |
| 6 | C=O | 1745 | (99) | 1742 | (100) |
| 7 | C=C | 1652 | (24) | — | — |
| 8 | | 1463 | (74) | 1464 | (39) |
| 9 | | 1416 | (47) | 1417 | (30) |
| 10 | | 1377 | (56) | 1378 | (36) |
| 11 | | 1239 | (71) | 1239 | (49) |
| 12 | C–C | 1163 | (89) | 1167 | (66) |
| 13 | | 1100 | (71) | 1098 | (47) |
| 14 | | 1029 | (38) | — | — |
| 15 | | 969 | (30) | 979 | (30) |
| 16 | | 915 | (28) | — | — |
| 17 | | 867 | (21) | — | — |
| 18 | | 794 | (17) | — | — |
| 19 | cis-alken | 722 | (61) | 725 | (13.5) |
| 20 | | 603 | (14) | — | — |

acid. Unlike fresh linseed oil which remained unaffected in the presence of NaOH marked concentrations of carboxylic acid groups of aged linseed oil (or linoxin) have reacted with the added NaOH attributable to the fact that 55 % of the sodium hydroxide solution were consumed (Fig.2).

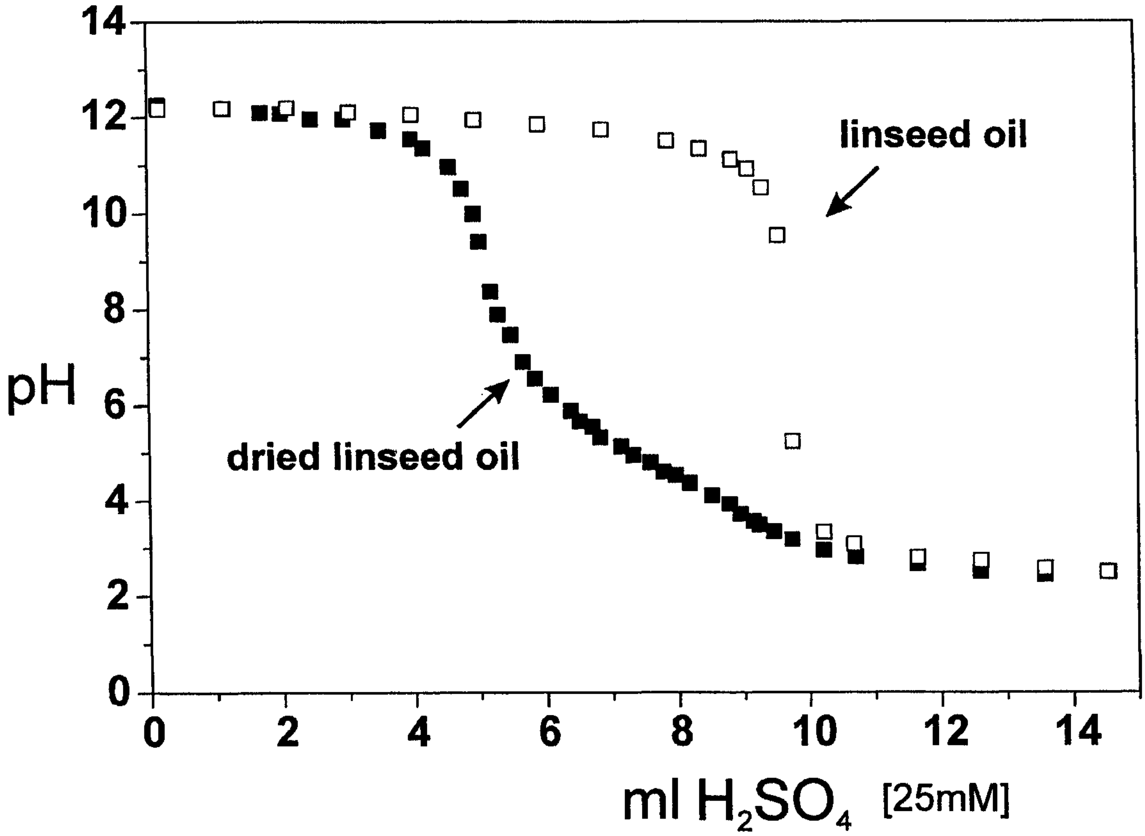

**Fig. 2:** Titration of saponified linoxin. Linoxin and linseed oil (84.1 mg each) were treated for 1 hour with 10.0 ml cold, aqueous 50mM sodium hydroxide. Back titration was performed with 25mM sulfuric acid. Linseed oil (□) results in the steep curve while a more shallow curve originates from linoxin (■). The amount of sulfuric acid used to titrate linseed oil is exactly equivalent to the amount of sodium hydroxide applied (steep drop of the pH value at the equivalence point 10.0 ml). Linseed oil was accordingly not saponified. The titration curve of linoxin drops steeply at 4.5 ml sulfuric acid. The difference to the theoretical amount of sulfuric acid required (10.0 ml) shows that 5.5 ml of the 50mM sodium hydroxide were consumed.

### 3.2. Varnish removal with lipase

In 1988 a promising new technique for the cleaning of painting surfaces was introduced employing lipase /2,3/. This new approach was repeatedly employed /6,7/ and critically examined /8,9/. By way of contrast to the classical methods based on the use of organic solvents, the application of mild and selective biochemical reagents was considered revolutionary. An exciting advantage over the earlier established procedures was seen in the highly specific removal of one type of varnish among many other different varnishes /10/. This new method was claimed to be both gentle and protective. In the initial approach aqueous gels in the presence of lipase previously dissolved in weak buffers were applied /2,3,11/. The advantage was that virtually insoluble oil varnishes applied over resin varnishes could be effectively removed. Pure resin based varnishes were expected to be resistant to the lipase treatment and to act as a barrier avoiding undesired reactions on lower layers /12/.

The efficacy of the enzymic cleaning system was examined using standardized test samples. Window glass plates coated with linseed oil were subjected to an oxidative drying process for 3 - 6 months. This pre-treatment was expected to ascertain the chemical relationship to historically aged oil varnishes in a reproducible and controlled manner. The varnishes were aged by heating the varnished plates to 40° for 3 - 6 months. These sample preparations under highly reproducible laboratory conditions were preferred to older paint and varnish layers of unknown and uncontrolled origin. A further important criterion was the use of chemically non reactive glass as a ground material. Any undesired reactions with the applied varnish could be excluded. For further experimental details see experimental section.

All cleaning suspensions were freshly prepared. The cleaning suspension is composed of two components. Component one contains aqueous 0.05 M Tris/HCl - buffer at pH 7.7-8.4 into which hydroxypropylmethylcellulose was added to obtain a gel of high viscosity. Triton X-100 was added to improve the wetting of the surface. Prior to use, the lipase was suspended as component two in the gel and applied onto the oil varnish. The gel was allowed to react for 12 min. The varnished surface of a glass plate was divided into five separate tracks each of which was differently pre-treated (Fig.3).

On tracks 1 - 4 a yellowish/brownish colour developed. The varnish layers became soft and gelatinous and increased considerably in volume. They were easily removed by cotton swabs. The cotton tipped plastic rods were gingerly hand rolled over the respective test track carefully avoiding any pressure on the tip. The varnish on track 5 (control gel) remained intact and could not be removed by a cotton swab.

Contrary to the assumptions on which the lipase formula is based, the same cleaning effect was observed when the lipase was omitted. Thus, the weakly alkaline Tris buffer must be assigned to be the actual reactive component responsible for the observed activity of the mixture (Fig. 3).

The Tris buffer base employed in the 'enzymic cleaning experiment' is known to be a powerful chelator of heavy metal ions. Pigments in contact with this buffer base can react to form complexes. Many of these compounds are exceedingly reactive and can substantially affect the painting even years after the initial treatment. When malachite oil paint (basic copper carbonate, green modification) is suspended in the Tris buffer solution used in the enzymic cleaning protocol, blue coloured Cu (II) -Tris complexes are formed (Fig.S1). In the presence of reducing systems aggressive oxygen species are detectable /13-17/ which are known to break down many biopolymers.

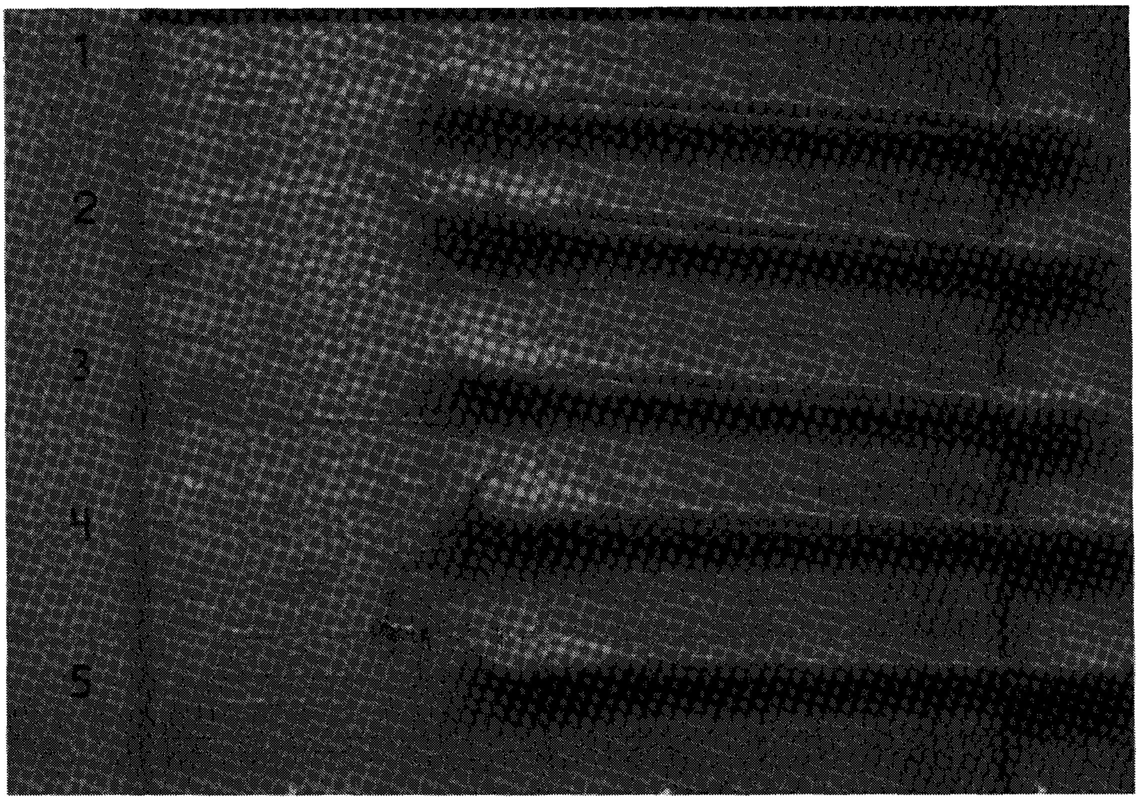

**Fig. 3:** Reactivity of lipase and Tris buffers on dried linseed oil varnish. Dried linseed oil on a glass plate after 20 minutes treatment with weakly alkaline buffer gels. The soft, deteriorated varnish can easily be removed by a cotton swab. The addition of lipase had no effect. At pH 8.4 the reaction is stronger than at pH 7.7.

1: buffer gel pH 7.7, omitting lipase
2: buffer gel pH 8.4, omitting lipase
3: buffer gel pH 7.7, in the presence of lipase
4: buffer gel pH 8.4, in the presence of lipase
5: control gel, omitting both buffer and lipase

### 3.3. The new concept: large $M_r$ solvents - strong bases - large counter ions.

Taking into account the effective removal of varnish by alkali hydroxides, this long known phenomenon was reconsidered in applying this reaction in a controlled manner. Alkaline varnish removal techniques are occasionally applied in the cleaning of paintings. In extreme cases, alcoholic solutions of sodium hydroxide are used to supplement the usual aqueous solutions of ammonia.

While the application of alcoholic sodium hydroxide to paintings may initially appear outrageous, the following considerations show that very strong bases may be far less dangerous than weakly alkaline buffer systems and may be employed in varnish removal if the following guidelines are observed:

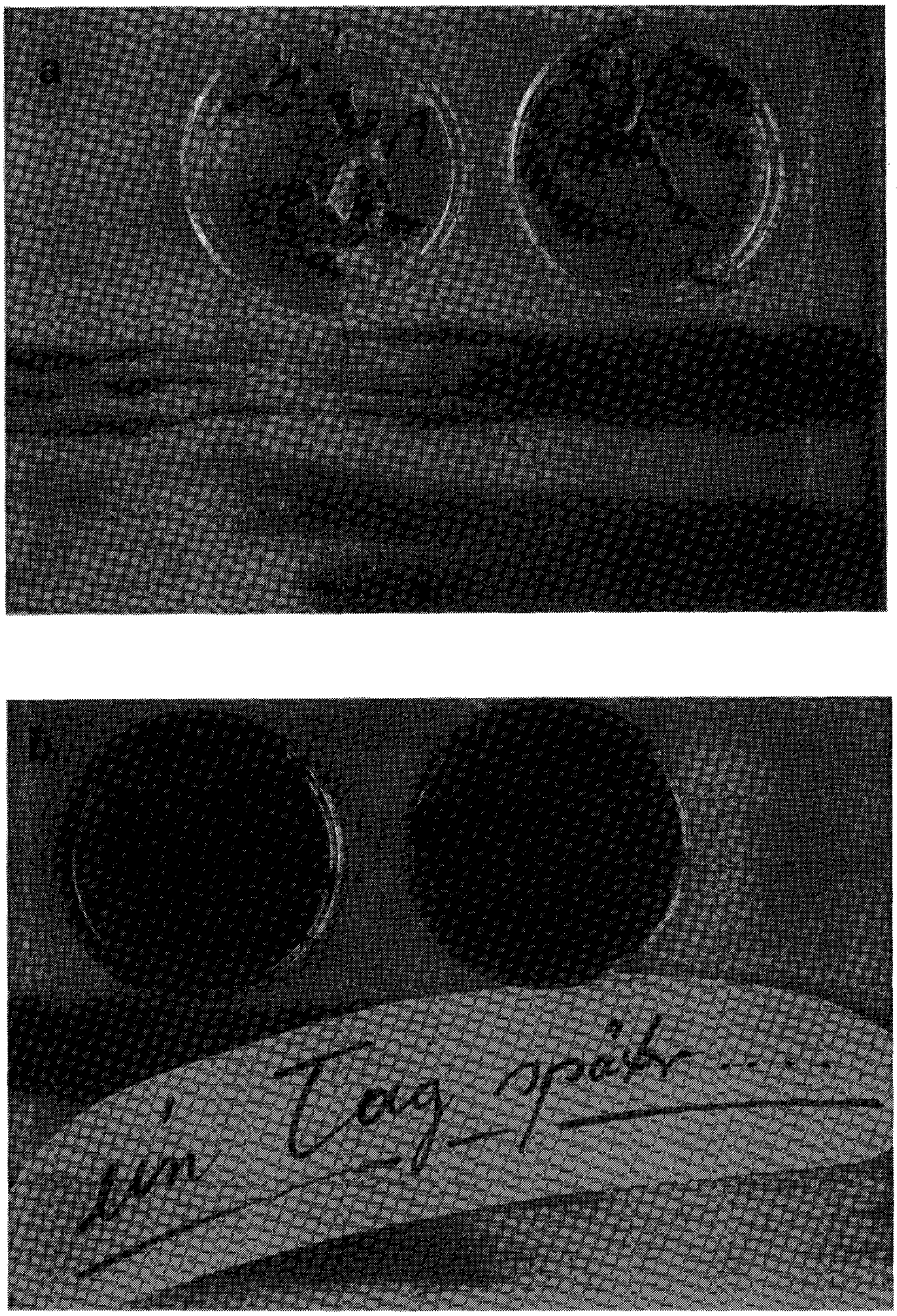

**Fig. S1:** Copper complex formation from mineral based pigments in the presence of Tris buffer.

a) Dried linseed oil paint samples containing copper pigments (dark green = verdigris; pale green = malachite). The samples were placed in a glass dish and suspended with aqueous 0.05 M pH 8.4 Tris buffer solution. Within 20 minutes the solution is colored light blue.

b) After one day the solution is colored deep blue due to the formation of Tris copper complexes.

***Rule 1:*** Use large relative molecular mass ($M_r$) solvents

Small $M_r$ solvents (water, ethanol etc.) cause varnish layers to swell and soften. As a result, the solvent can penetrate the varnish layer and cause severe damage to the paint layers below. Consequently, large $M_r$ solvents should preferably be used for restoring paintings.

***Rule 2:*** Low concentrations of strong bases are safer than high concentrations of weak bases

Very dilute solutions of strong bases are alkaline enough to remove varnish. When applied to varnish layers, these dilute solutions react quickly and are consumed at the surface layer. They, therefore, lack the time to penetrate into deeper layers. Strong bases also hydrolyse varnish ester groups practically quantitatively, effectively eliminating the applied base at the same time - an additional advantage.
Weak bases do not hydrolyse varnish completely. In buffered solutions, the presence of their corresponding acids reduces their efficacy even further. Therefore, weak bases or buffers must be applied in high concentrations to achieve comparable effects. However, varnish hydrolyses much slower in weak alkaline solutions than in strong alkalines, allowing the small buffer ions more time to penetrate lower layers and cause damage. The $OH^-$ ion is a very strong base. Accordingly, dilute solutions of sodium hydroxide are perfectly suitable for the removal of aged varnish.

***Rule 3:*** Employing large $M_r$ cations.

The application of sodium hydroxide. or ammonia should, however, be avoided as hydroxide ions may diffuse into lower paint and varnish layers. This diffusion of hydroxide ions is possible if they are accompanied by small, diffusible counter ions.

Due to their permeability, varnish layers can be viewed as a 'cation sponge'. Invading hydroxide ions hydrolyse the ester groups and the accompanying cations neutralize the resulting carboxylate groups. Consequently, small cations should be avoided wherever possible in the restoration of paintings. This rule should not only be observed when working with strong bases such as hydroxide ions but also in cases where weak bases are employed to neutralize acids. As such, the damaging effect of desoxycholate /18/ or resin detergents /19/ can be assumed to be primarily due to penetration of small cations into the binder and its consequent hydrolysis.

Accordingly, the application of these 'mild' detergents in the conservation of paintings should be avoided. The only exception should be cases in which neither of the layers (varnish, paint or base) contains hydrolysable ester components. Exchanging small cations for large ones practically 'docks' the hydroxide ions onto the surface of the painting.

### 3.4. The application of high $M_r$ alkali crown ether

Appropriate solvents are the polar polyethylene glycol-400 (PEG-400) or the less polar polypropylene glycol. Large $M_r$ alcohols such as dodecanol can be employed as well. Large countercations prevent hydroxide ions from penetrating into lower paint layers. This has the desirable effect of limiting the effect of hydroxide ions to the surface of the varnish. In non-aqueous solvents, other anionic bases may be employed as well. In this context our interest fell on PEG-400 and alkali hydroxides. PEG-400 is not only a large $M_r$

solvent but also has excellent complexing characteristics, e.g. for alkali metal ions /20.. In this context it should be kept in mind that in biological systems many crown ether type complexes are formed, for example, Rb-nonactin (Fig 4)

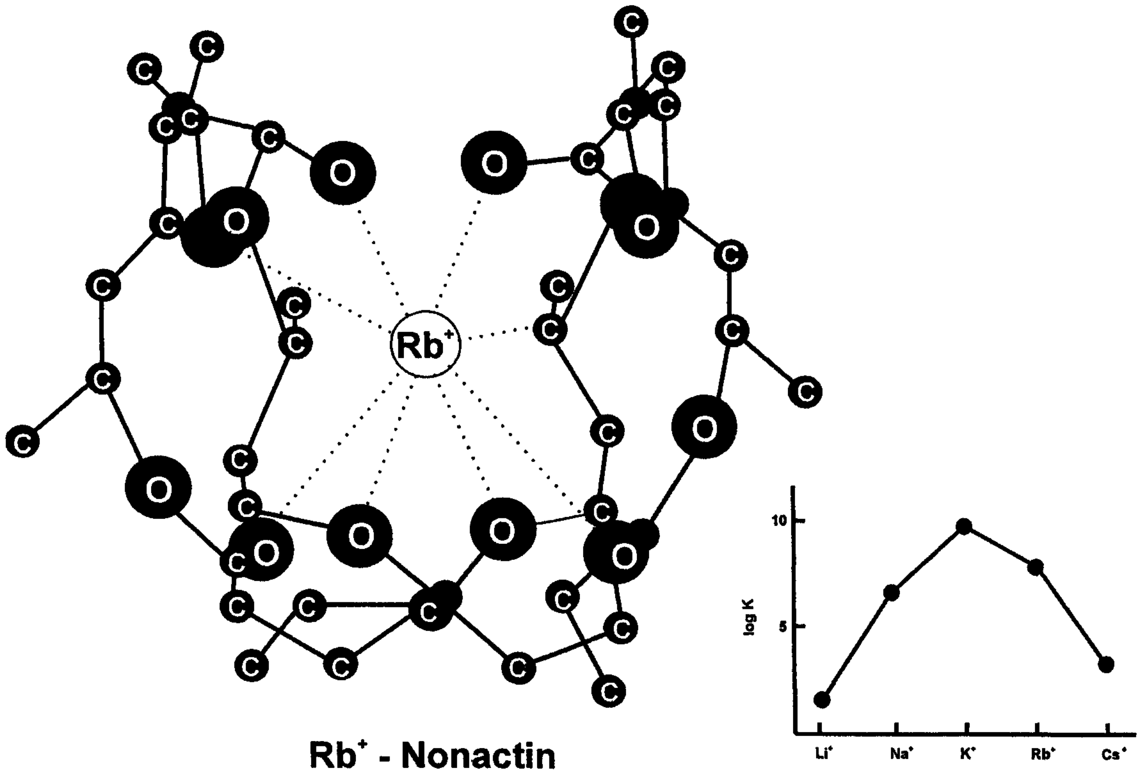

**Fig. 4:** The structure of Rb - Nonactin, an example of a biological alkali ion crown ether type complex. *Lower right side:* Stability constants of alkali-ion cryptates, redrawn after ref. /24/.

In a similar way, large complexes resembling crown ethers are formed when alkali metal hydroxides, alcoholates, etc. are dissolved in PEG-400 /21/, which encircles an alkali metal cation (Fig 5). The ionic radius determines the resulting ring size. The most stable alkali ion crown ethers were obtained in the presence of potassium ($K^+$) followed by $Rb^+$, $Cs^+$, $Na^+$ and $Li^+$ /22/ (Fig 5). The ring size of the macro cyclic metal complex is determined by the size of the alkali metal ion radius. This phenomenon is called template effect /22,23/.

The genuine formation of a Rb-PEG-400 complex was demonstrated by passing a mixture of RbOH and PEG-400 through a Bio-Gel P-2 column. It was clearly shown that the Rb-ions remained firmly bound in the polyethyleneglycol fractions. The stability of the *in situ* generated crown ether type complex was surprisingly high to survive such a long range gel filtration (Fig 6). The major portion appears slightly shifted directly under the shoulder of the PEG-400 absorption at $A_{210\,nm}$. Absolutely no dissociated $Rb^+$-ions were detected in front or after the PEG-400 absorption band supporting the conclusion of a high stability.

Of special importance was the use of $Rb^+$ as in a later stage the radioactive $^{86}Rb$ ion was applied to study possible migration into the lower paint layers. Thus, this cleansing regime in the presence of $Rb^+$ was documented in more detail (Fig.7).

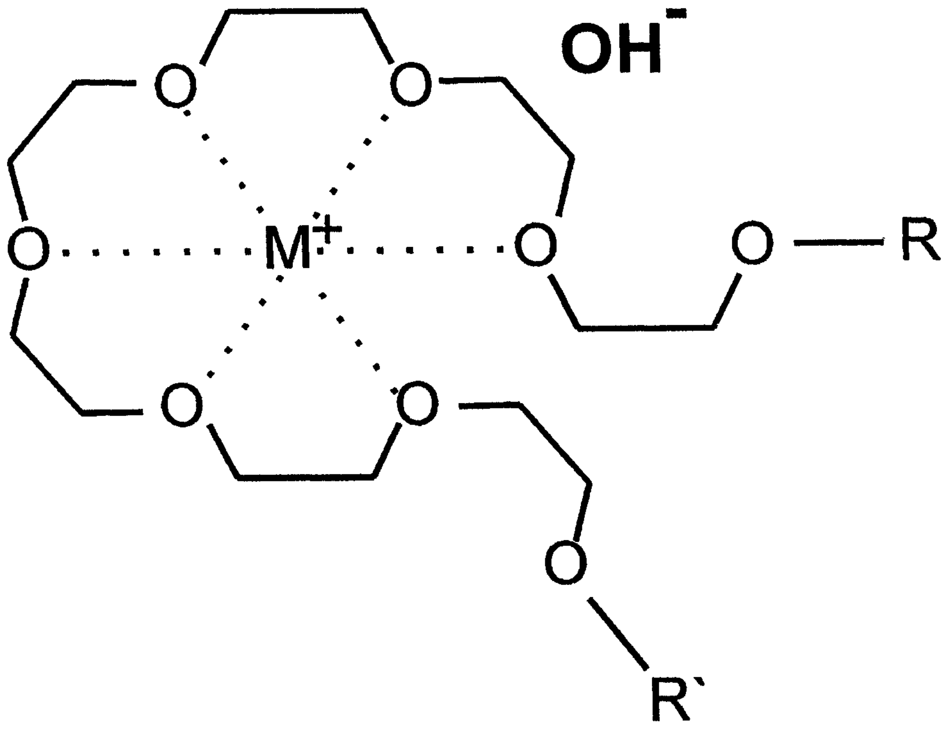

**Fig. 5:** The structure of alkali-ion ($M^+$) polyethylene glycol-400 (PEG-400) crown ether.

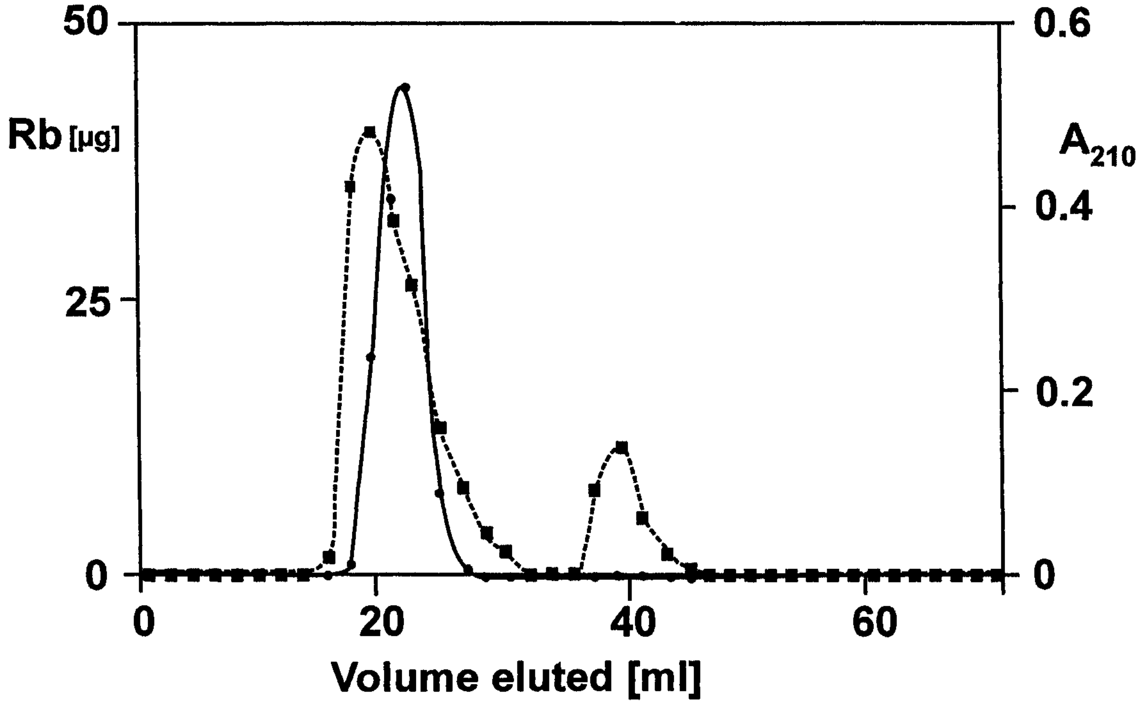

**Fig. 6:** Gel chromatography of *in situ* formed RbOH-PEG-400 crown ether type complex. - ■ - $A_{210\ nm}$ ; - ● - Rb(I). 10 ml of a mixture of 1 mol/l RbOH and a 28 fold excess of PEG-400 were passed through Bio-Gel P-2 column. No detectable dissociation of the RbOH-PEG-400 complex was seen supporting the conclusion of a high stability of this complex.

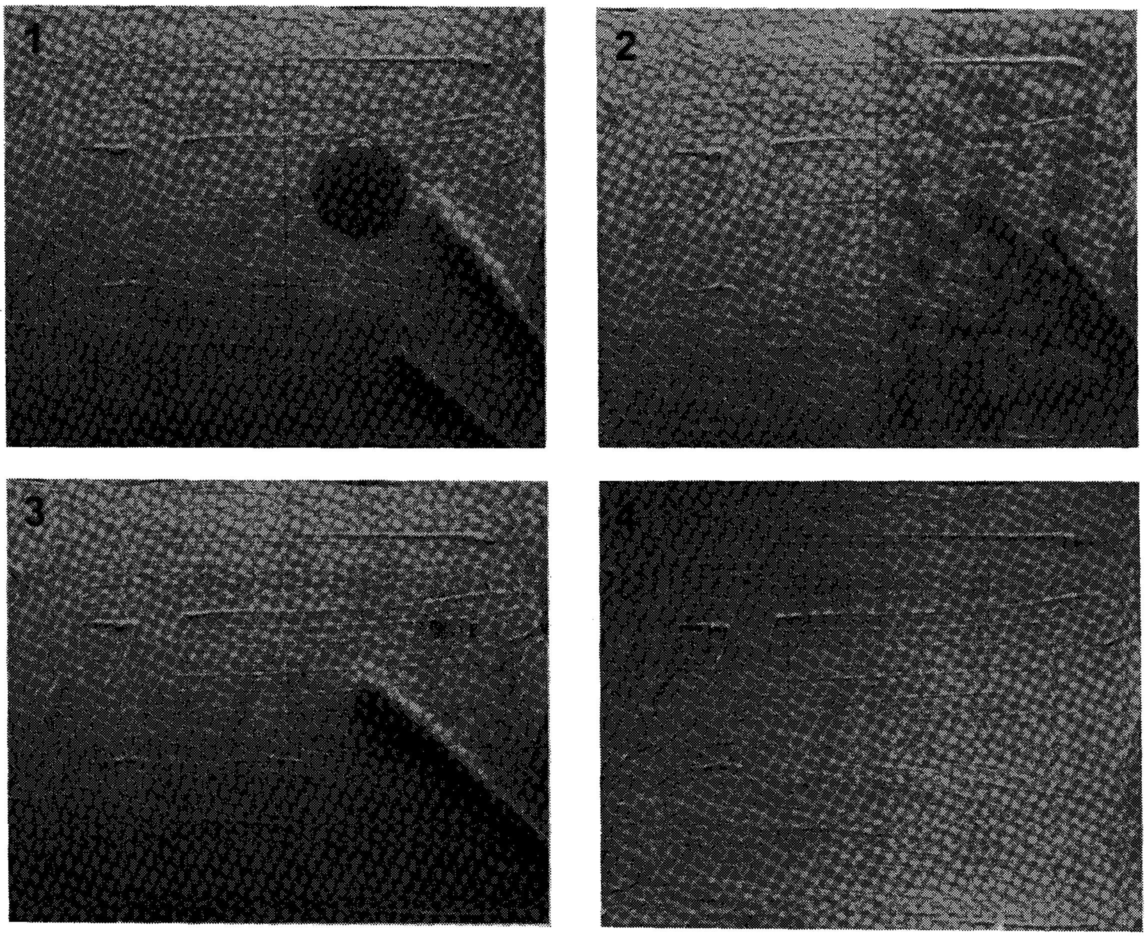

**Fig. 7:** Detailed steps of RbOH - PEG crown ether varnish removal: The test surface is a dried paint layer consisting of lead white - linseed oil. The paint layer is covered with a dried, approximately 7 years old varnish layer of linseed stand oil. The RbOH / PEG cleanser is applied by gingerly moving a cotton swab or a polypropylene spatula over the surface. The brownish color of the RbOH / PEG - crown ether is progressively intensified after an incubation of 2 minutes. The cleanser containing dissolved varnish components is removed mechanically. The cleansed surface is subsequently neutralized with polyacrylic acid / PEG-400 and consecutively treated using large $M_r$ solvents of decreasing polarity. In the final step, the surface is treated with Shellsol T and allowed to evaporate. Following the successful removal of the yellowed varnish, the cleansed surface is noticeably lighter. *Important notice*: No detectable abrasion of the paint surface is microscopically seen after this treatment!

Varnish removal is slower as compared to aqueous or alcoholic solutions of the same concentration. Fortunately, this PEG-mixture is allocated to the varnish surface.

Varnish removal is especially slow if the PEG-400 solution is not disturbed once applied to the surface of the painting. The high viscosity, as well as, the large $M_r$ reduces diffusion and convection rates of varnish hydrolysis products. Accordingly, the restorer can determine the rate of varnish removal by gentle movement of the reaction solution (e.g. with a cotton swab or a polypropylene spatula). Oil varnish cleavage products dissolve in PEG-400, coloring it orange-brown.

Removal of this solution terminates the reaction when the desired degree of cleansing has been achieved. The remaining reaction solution is removed in subsequent stepwise washes. The advantage of this varnish removal technique over conventional methods is that varnish is hydrolysed only at its surface. As such, the effect of this technique resembles that of a 'chemical plane'. Varnish can be removed or thinned even if the varnish and base are chemically similar (e.g. oil varnish on an oil paint). After the cleansing process no detectable abrasion or levelling off of the paint surface was noticed both visually and after microscopic examination. Detailed steps of varnish removal with the RbOH - PEG crown ether method are depicted in Fig.7.

### 3.5. Application of RbOH/PEG 400 crown ether for varnish removal on Old Master paintings

The new method was demonstrated on two fragments of Old Master paintings.

A canvas fragment of a late 17th century oil painting was cleansed during a workshop at the Courtauld Institute of the History of Art, London. The picture varnish was of a deep brown color. The fresh prepared RbOH / PEG-400 cleanser was applied onto the varnish surface without craquelures under slight agitation using a polypropylene spatula.

This varnish most likely was of a polyester structure as its removal succeeded in a smooth manner. After 3-4 minutes the treatment was terminated by the removal of excessive RbOH / PEG-400 employing dry cotton swabs. The cleansed surface was subsequently neutralized with polyacrylic acid / PEG-400 and repeatedly washed with large $M_r$ solvents of decreasing polarity. In the end the de-varnished area was of exceptional quality (Fig 8).

The 19th century oil painting 'Die Fischhändlerin' ('pseudo Frans Hals') was treated on a small section center left at the Institut für Restaurierungs- und Konservierungs-wissenschaften, Köln. After employing the RbOH/PEG-400 crown ether method on the aged varnish, the dark-greenish-brown colored sky of the original painting emerged in its original bright blue color (Fig.S2). No detectable abrasion of the paint surface was microscopically seen in either cleaned paint fragment after this treatment!

### 3.6. Possible absorption of RbOH/PEG-400 crown ether and polyacrylate onto the paint surface

In order to locate possible absorption of $Rb^+$-ions in the course of alkaline varnish removal, the cleansing solution was enriched with $^{86}Rb$. This radioactive isotope was chosen attributable to the short half-life of 18.6 days avoiding thereby possible damage of the paint ground due to uncontrolled irradiation.

In order to compare the efficacy of the RbOH / PEG-400 crown ether, cleansing solutions of both ethanolic and aqueous $Rb(^{86}Rb)OH$ were applied. Already mere mechanical wiping leaves only 2.5 % of $^{86}Rb$ on the paint surface. In the follow up treatment the Rb-PEG-400 cleanser and its removal with polyacrylic acid in PEG-400 reduces the rubidium ion surface concentration to 0.8 % of its initial amount. By way of contrast a thirty-fold higher $^{86}Rb$ radioactivity (23.3 %) was seen after the ethanolic $Rb(^{86}Rb)OH$-treatment. The use of aqueous $Rb(^{86}Rb)OH$ resulted in an almost 40-times higher residual $^{86}Rb$ radioactivity (30.1 %)

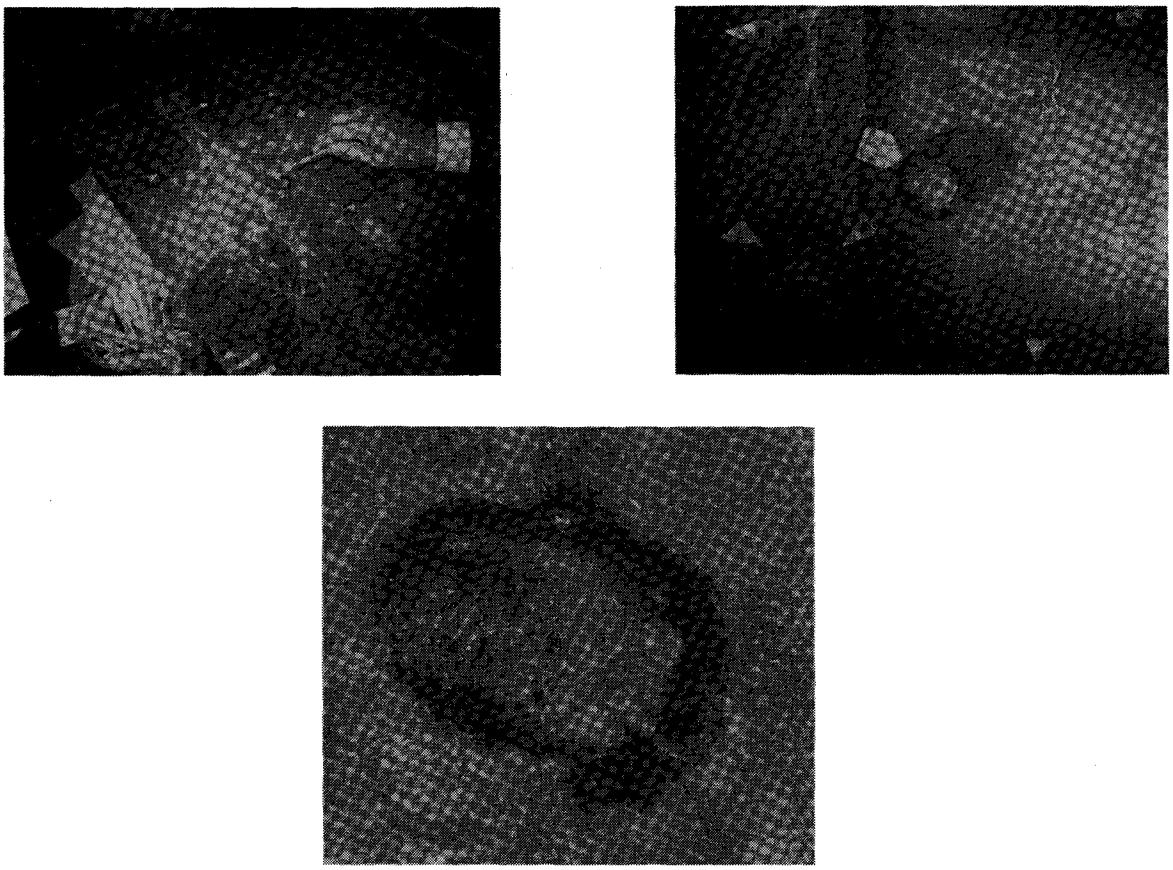

**Fig. 8:** Practical application of RbOH / PEG-400 on a late 17th century oil painting. The new method was demonstrated during a workshop at the Courtauld Institute in London compared to the crown ether treatment (Table 2).

In the case of $^{14}C$-polyacrylic acid neutralisation experiments aiming at possible surface absorption of $^{14}C$-polyacrylate the post cleansing process was carried out as described in *2.2.3.* No $^{14}C$-polyacrylate was detected at the end of the cleansing process indicating that no absorption of this viscous poly-acid to the paint surface was noticed.

### 4. CONCLUSION

### 4.1. The enzymic approach for varnish removal

A genuine reactivity of lipase on aged linseed oil varnish was not seen. Lipases are of large relative molecular mass, between 40 000 and 60 000 Daltons. In the active centre which represents only a minor portion of the molecule the fatty acid esters are cleaved. Lipases are insoluble in oil and are active exclusively at the lipid/water interface. A detectable activity is seen when lipids are finely emulsified leading to a substantial enlargement of the two adjacent surface areas. The surface of a paint film cannot be enlarged by emulsification. The reaction is dependent on water, but excessive water cannot be used as linoxyn

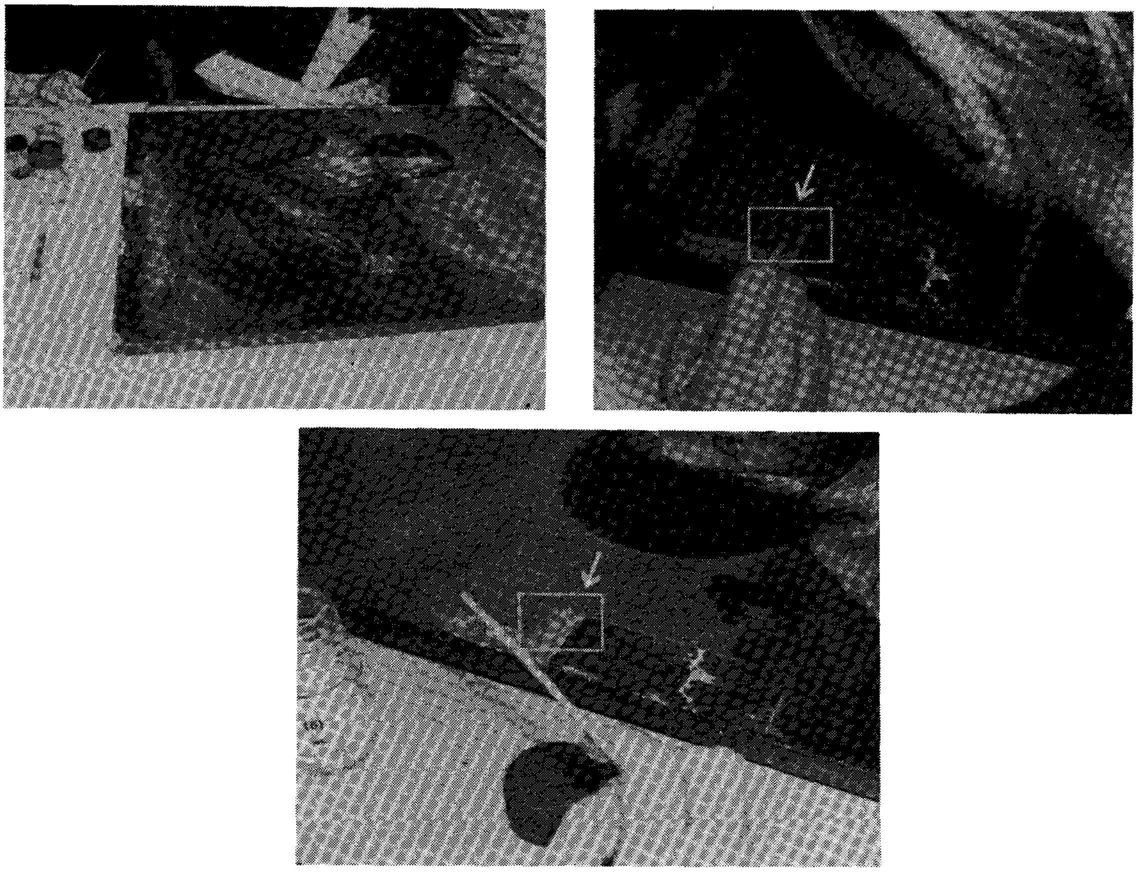

**Fig. S2:** Practical application of RbOH / PEG 400 on the 19th century oil painting 'Die Fischhändlerin'. Overpainted "pseudo Frans Hals" 1874. Workshop FH-Köln 2002.

substantially swells and deteriorates. Prolonged exposure to water can damage both the paint layers and the ground.

A successful enzymic cleaning would lead to considerable concentrations of the reaction products including glycerol and fatty acids, which must be removed. Oligomeric forms of fatty acids are soluble in water at a pH range between 12-13 and virtually insoluble at lower pH values. High pH-values are too alkaline for use on oil media. Thus, it is highly questionable that the enzymic cleavage of oil varnish layers was possible under the given conditions.

In conclusion, a number of questions have been raised regarding the activity and safety of lipase mixtures used to remove oil varnishes. According to the evidence presented here we leave it to the conservator to decide whether or not polar aqueous media should be used for the removal of paint films. The small molecular weight water molecules have been found to swell both varnish and paint layers especially when weak alkaline buffers are carried into deeper regions where they can exert the reported activities leading to deteriorating oxidase-mimeting transition metal complexes. It should be emphasized that lipase is not reactive under the given conditions. All the observed reactivities are assigned to the Tris-buffer.

The challenge for both conservators and chemists remains. An alternative approach was the application of alkaline crown ether for the removal of aged varnishes.

**Table 2**

Residual rubidium ions on paint surface

| $Rb(^{86}Rb^{+})$-applied [2.4 *μmol/cm²*] = 900 cpm | radioactivity [*cpm*] | $Rb(^{86}Rb^{+})$ [*μmol/cm²*] | Residual $Rb(^{86}Rb^{+})$ [%] |
|---|---|---|---|
| $Rb(^{86}Rb^{+})$-PEG-crownether *wiping off* | 23 | 0.06 | **2.5** |
| $Rb(^{86}Rb)$-PEG-crownether *post-cleansing* | 8 | 0.02 | **0.8** |
| $Rb(^{86}Rb^{+})$-ethanol *wiping off* | 850 | 2.23 | **94.5** |
| $Rb(^{86}Rb^{+})$-ethanol *post-cleansing* | 210 | 0.55 | **23.3** |
| aqueous $Rb(^{86}Rb^{+})OH$ *wiping off* | 380 | 0.99 | **41.9** |
| aqueous $Rb(^{86}Rb^{+})OH$ *post-cleansing* | 280 | 0.73 | **30.9** |

Mere mechanical wiping already leaves only a small amount of $^{86}Rb$ (2.5 %) on the paint surface. By way of contrast, most of the alcohol-based cleanser evaporated on the varnish surface. Consequently, practically all of the applied rubidium remains on the surface leaving (94.5 %) of residual reactivity. Still some 42 % of $^{86}Rb$ were measured on the surface after a water-based cleansing process.

### 4.2. Application of alkali-crown ether for the removal of varnish; the applied bioinorganic chemistry approach

The alkali-crown ether induced removal of varnishes of polyester type is a promising new method in the conservation of cultural heritage. Nevertheless the application of this method should be performed with great care. Alkaline removal of varnish should, consequently, be restricted to aged, oil-based varnishes and oil paintings. In any case, preliminary tests must be conducted on an unobtrusive area to confirm applicability. Oil varnishes covering very thin layers of oil paint or paint varnishes should not be removed completely by this method. Varnish thinning should be preferred as the method of choice. Removal and/or thinning of later over paint based on linseed oil may be recommended. Of course, extended microscopic and electron micrographic controls will be necessary. The result of the application of this new technique on genuine paintings was promising. However, long-time observations of this cleaning process are still necessary to find whether or not any hidden damage may emerge. The alkaline removal technique should also not be applied in cases of deep craquelures or when the paint layer is peeling off concomitantly uncovering the ground. Thus, alkaline solutions could creep under the surface attacking or separating the pigment containing ground.

### ACKNOWLEDGEMENTS

The generous support of the Landesbank Baden-Württemberg, Stiftung Kunst und Kultur, Germany, 'Firnisuntersuchung an Gemälden' File No.: 200 1040129 is gratefully appreciated. Dr.Aviva Burnstock, Courtauld Institute of the History of Art, London, and Prof. Hans Portsteffen, Institut für Restaurierungs- und Konservierungs-wissenschaften, FH-Köln for kindly providing authentic fragments of 18th and 19th century paintings. Thanks go to Dr. Raymond White, Department of Scientific Research, National Gallery London and Dres. Hans-Jürgen Hartmann, Albrecht Jung, Yoka Kaup and Christian-Heinrich Wunderlich, Physiologisch-Chemisches-Institut, Universität Tübingen, for many stimulating discussions. The generous donation of $^{14}$C polyacrylic acid by Dr. Eugen Barbu, School of Pharmacy and Biomedical Sciences, University of Portsmouth, Hampshire, UK is gratefully acknowledged.

### ABBREVIATIONS

| | | |
|---|---|---|
| PEG-400 | = | polyethylene glycol 400 |
| RbOH / PEG-400 | = | rubidiumium hydroxide in PEG-400 |
| $M_r$ | = | relative molecular mass |

### REFERENCES

1. E. Berger, Quellen und Technik der Fresko-, Oel- und Tempera-Malerei des Mittelalters, von der byzantinischen Zeit bis einschließlich der Erfindung der Oelmalerei durch die Brüder van Eyck, 3.Folge, München, 1912, pp.15-83.
2. R. Wolbers, American Institute for Conservation preprints, 251-280 (1988).
3. R. Wolbers, *Notes for a Workshop on New Methods in the Cleaning of Paintings*, Getty Cons. Inst. (1990).
4. H.P. Kaufmann, *Fette, Seifen, Anstrichmittel* **59**, 153- 162 (1957).
5. M. Lazzari, O. Chiantare, *Polym. Degr. Stab.* **65**, 303 – 313 (1999).
6. M. Hebeisen, *Restauro* **1**, 18-25 (1991).
7. F. Hellwig, *Arbeitsblätter für Restauratoren* Gr.12 Möbel 2 71-74 (1991).
8. B. von Gilsa, *Zeitschrift für Kunsttechnologie und Konservierung* **5**, 48-58 (1991).
9. C.H. Wunderlich, U. Weser, *Restauro* **101**, 22-27 (1995).
10. P. Geusau, M. Schreiner, *Zeitschrift für Kunsttechnologie und Konservierung* **6**, 260-274 (1992).
11. U. Weser, C.H. Wunderlich, *Zeitschrift für Kunsttechnologie und Konservierung* **10**, 147-152 (1996).
12. R. Wolbers, unpublished protocols, workshop Köln 1992.
13. U. Deuschle, U. Weser, *Prog. Clin. Biochem. Med.* **2**, 97-130 (1985).

14. J. Müller, K. Felix, C. Maichle, E. Lengfelder, J.Strähle, U. Weser, *Inorg. Chim. Acta* **233**, 11-19 (1995).
15. L.O. Klotz, J. Müller, M. Fausel, R. Gebhard, U. Weser, *Biochem. Pharmacol.* **51**, 919-929 (1996).
16. J. Lange, H. Elias, H. Paulus, J. Müller, U. Weser, *Inorg. Chem.* **39**, 3342-3349 (2000).
17. J. Müller, D. Schübl, C. Maichle-Mössmer, J. Strähle, U. Weser, *J. Inorg. Biochem.* **75**, 63-70 (1999).
18. J. Koller in: J.S. Mills, P. Smith (Eds.), *Cleaning of a Nineteenth-Century Painting with Deoxycholate Soap: Mechanism and Residue Studies.* Brussels Congress on Cleaning, Retouching and Coatings. Technology and Practice for Easel Paintings and Polychrome Sculpture. IIC preprints Brussels, 1990, pp.106-110.
19. B. von Gilsa, *Zeitschrift für Kunsttechnologie und Konservierung* **1**, 184-218 (1993).
20. G. Illuminati, L. Mandolini, B. Masci, *J. Am. Chem. Soc.* **105**, 555 – 563 (1983).
21. L. Mandolini, B. Masci, *J. Am. Chem.Soc.* **106**, 168 – 174 (1984).
22. C. A. Vitali, B. Masci, *Tetrahedron* **45** (7), 2213 – 2222 (1989).
23. S. Chun, S.V. Dzyuba., R.A. Bartsch, *Anal. Chem.* **73** (15) 3737-3741 (2001).
24. J.J.R.F. da Silva, R.J.P. Williams, *The Biological Chemistry of the Elements*, Clarendon, Oxford, 1991, p. 39.

